# Developmental, Morphological and Physiological Traits in Plants Exposed for Five Generations to Chronic Low-Level Ionising Radiation

**DOI:** 10.3389/fpls.2020.00389

**Published:** 2020-04-15

**Authors:** Nicol M. Caplin, Alison Halliday, Neil J. Willey

**Affiliations:** Centre for Research in Biosciences, Faculty of Health and Applied Sciences, University of the West of England, Bristol, United Kingdom

**Keywords:** ionizing radiation, chronic exposure, Derived Consideration Reference Level, radiation biology, phenotype

## Abstract

The effects of ionising radiation (IR) on plants are important for environmental protection but also in agriculture, horticulture, space science, and plant stress biology. Much current understanding of the effects of IR on plants derives from acute high-dose studies but exposure to IR in the environment frequently occurs at chronic low dose rates. Chronic low dose-rate studies have primarily been field based and examined genetic or cytogenetic endpoints. Here we report research that investigated developmental, morphological and physiological effects of IR on *Arabidopsis thaliana* grown over 7 generations and exposed for five generations to chronic low doses of either ^137^Cs (at a dose rate of *c.* 40 μGy/h from β/γ emissions) or 10 μM CdCl_2_. In some generations there were significant differences between treatments in the timing of key developmental phases and in leaf area or symmetry but there were, on the basis of the chosen endpoints, no long-term effects of the different treatments. Occasional measurements also detected no effects on root growth, seed germination rates or redox poise but in the generation in which it was measured exposure to IR did decrease DNA-methylation significantly. The results are consistent with the suggestion that chronic exposure to *c.* 40 μGy/h can have some effects on some traits but that this does not affect function across multiple generations at the population level. This is explained by the redundancy and/or degeneracy between biological levels of organization in plants that produces a relatively loose association between genotype and phenotype. The importance of this explanation to understanding plant responses to stressors such as IR is discussed. We suggest that the data reported here provide increased confidence in the Derived Consideration Reference Levels (DCRLs) recommended by the International Commission for Radiological Protection (ICRP) by providing data from controlled conditions and helping to contextualize effects reported from field studies. The differing sensitivity of plants to IR is not well understood and further investigation of it would likely improve the use of DCRLs for radiological protection.

## Introduction

Ionizing radiation (IR) regulations, long focussed primarily on humans, have only relatively recently considered effects on the environment, including flora and fauna (ICRP, 2003). This has developed into a recommended system of radiological protection based on reference animals and plants (ICRP, 2008) and regulatory thresholds for them (ICRP, 2014). Some key regulatory thresholds for dose rates to plants have been questioned because of a variety of effects reported from a range of dose rates and at different levels of biological organization (Hayashi et al., 2015; Møller et al., 2016; Oladosu et al., 2016) and there is still significant controversy over the effects of radiation in the environment at, for example, Chernobyl and Fukushima (Beresford et al., 2020). In an era when reductions in anthropogenic CO_2_ emissions to the atmosphere are vital for minimizing humanity’s effects on global climate and in consequence several nations are investing in nuclear power as a ‘low-carbon’ electricity source, the balance that IR regulations must strike between environmental risks and benefits is the focus of significant scrutiny. In addition to the 452 operating nuclear reactors whose impact on the environment must be regulated (IAEA, 2019), there are 54 more under construction. Regulators must also oversee the environmental impact of decommissioning the 173 permanently shut down reactors and the construction and management of repositories (which is only in the early stages) for all the high-level nuclear waste ever generated. Incidents at nuclear facilities such as those at Khyshtym (in 1957), Windscale (1957), Chernobyl (1986), and Fukushima (2011) produced significant environmental contamination, much of which is still of environmental concern and is a reminder that regulation is also vital in the aftermath of nuclear incidents, including those that might arise from terrorism or the use of nuclear weapons.

Knowledge of the effects of IR on plants is, however, not just vital for regulating the nuclear industry and responding to nuclear incidents, it is also useful for plant science including agriculture, horticulture and plant stress biology (Caplin and Willey, 2018), space science (Arena et al., 2012; Arena et al., 2019) and to current developments in radiobiology (Kirsch et al., 2018). There has been much significant research into the effects of acute high doses of radiation at a variety of biological levels (e.g., Gicquel et al., 2011; Arena et al., 2017) so they are better understood than those of chronic low doses (Caplin and Willey, 2018). This is, in significant part, because of the widespread use of IR as a physical mutagen in agriculture and horticulture and to sterilize food products (Roberts, 2014). For plant science this is unfortunate, not only because chronic low doses are most relevant to the regulation of routine releases from nuclear sites but also because they are most relevant for understanding; (1) the role that ancient background radiation might have had in the evolution of plant stress responses, (2) for growing plants in space, and (3) complementing recent radiobiology studies on low level radiation and human health (Vaiserman et al., 2018). In 1996 a threshold dose rate of 417 μGy/h was recommended for radioprotection of plants (United Nations Scientific Committee on the Effects of Atomic Radiation [UNSCEAR], 1996) because effects on populations were not thought to occur below this level. Currently, the Derived Consideration Reference Levels (DCRLs) of the International Commission on Radiological Protection (ICRP) for chronic dose rates to reference terrestrial plants are *c.* 4.17 – 41.7 μGy/h for ‘pine tree’ (essentially to represent sensitive woody plants) and *c.* 41.7–417 μGy/h for ‘grass’ (essentially to represent less sensitive herbaceous plants). These DCRLs are dose rate ranges in which it is recommended that regulation should be considered because effects on communities and populations are thought to be possible. A species-sensitivity analysis based on the FREDERICA database suggested a 10 μGy/h screening dose rate for any ecosystem (Brown et al., 2008) but was intended to cater for organisms long-known to be more sensitive to IR than higher plants.

In the geological past environmental background dose rates from β/γ radiation probably reached a global average of almost 1 μGy/h but with considerable spatial variation – current global average background dose rate is *c.* 0.35 μGy/h with a maximum of about 15 μGy/h (132 mSv per annum) near Ramsar in Iran (Mortazavi et al., 2002) – so in the past maximum background may, in places, have been many times higher than 1 μGy/h. Inside the International Space Station dose rates are about 10 μGy/h depending on exact position (National Aeronautics and Space Administration [NASA], 2019), which are therefore the dose rates that food plants grown during space flight might experience (though locations in space craft or on planetary surfaces with less protection from cosmic radiation will experience higher dose rates). Much of the basis for the long-term radiological protection of humans is the data from high-dose acute exposure to radiation from atomic bomb detonations but several studies of chronic low-dose exposure of workers to radiation have been used to suggest adverse health impacts that are not necessarily predicted from acute exposure. For example, although many effects in such datasets can be driven by a few high exposure values, a dose rate of 2 μSv/h over 30 years has been suggested to produce an accumulated dose that can produce medically significant effects (Little et al., 2012). There are, therefore, many reasons for understanding the effects of chronic low-level radiation on plants, including understanding the effects of chronic exposure to IR in all living systems.

The majority of data on the effects of chronic low-level radiation on plants is from radioactively contaminated sites (Caplin and Willey, 2018), primarily those contaminated from nuclear incidents. Field data has particular strengths and weaknesses, and we suggest that this is especially important for studies of exposure to chronic low-level IR. In the case of chronic low-level radiation, correlating the values of measured traits in plants with dose rates at contaminated sites or comparing them with effects at ‘control’ sites has been widely used to make general statements about the relationship between dose rate and effects (Beresford et al., 2020). At Chernobyl, for example, it is challenging to establish cause and effect for such data for the following reasons; (a) in the contaminated areas around the Chernobyl nuclear power plant because of the short half-life of much of radioactivity released dose rates in the immediate days and weeks after the accident were very much higher than they have been in subsequent years (Hinton et al., 2007) and it is a difficult task to disentangle the effects of the unique acute high doses of radiation experienced by organisms in the past at Chernobyl from those produced later by exposure to chronic low dose rates (the effects of acute high doses of radiation on plants are heritable), (b) the ‘exclusion zone’ is now environmentally unusual not just in the presence of radioactivity, (c) establishing control sites that are identical but uncontaminated is genuinely difficult (most environmental variables can change on small spatial scales so even nearby ‘controls’ can be significantly different in many ways), and (d) measurements of the relevant traits in plants at sites before radioactive contamination occurred, i.e., before the ‘treatment’ occurred, are infrequent. And, it is also challenging to provide accurate dose rate measurements for plants in the field, especially in environments in which deposition of radioactivity has been spatially heterogenous and can include a significant number of, often highly radioactive, particles of various sorts.

Laboratory data generated under controlled conditions has significant weaknesses, especially in not being able replicate the complexity of the real field sites that have to be regulated and managed with all their idiosyncrasies but it can, at least to some extent, be complementary to field data through enabling an improved control over dose, dose history, and interacting environmental variables. The predominance of data from a few sites in chronic low-level radiation studies of plants reflects the urgency of understanding and managing contaminated sites but in order to strengthen the understanding of the general principles of the effects of IR on plants we suggest that more data from controlled conditions are necessary. DCRLs are intended to be a general starting point for regulation, and insights from radiation studies for plant stress biology and space science will be most useful if they are also generally applicable – so data from plants exposed to chronic low-levels of radiation under controlled conditions is a desirable complement to that generated in the field.

The most commonly measured endpoints in studies of the effects of ionizing radiation on plants are genetic, with measures of effects on DNA damage/repair, other cytogenetic effects and DNA methylation making up 75% of all studies in compilations of recent literature (Caplin, 2019). However, genotype and phenotype are only loosely coupled (Stearns and Hoekstra, 2000) and effects on the genotype are primarily of importance only when they affect the phenotype because it is generally the phenotype that is subject to natural selection and that determines the fitness of an organism. The loose coupling of genotype with phenotype produces a high level of redundancy and/or degeneracy, i.e., there can be significant changes or differences in genotype that do not affect phenotype (changes in gene sequence do not necessarily change gene products, mutations can inactivate genes or change gene products but there are often other copies of the gene that can make the product and so on), and the same genotype can produce different phenotypes because different environmental variation can produce different responses in an organism. ROS production/antioxidant capacity are the commonest measures of IR-induced stress (Smith et al., 2012) and a reminder that there can also be physiological buffers between genotype and phenotype. These are key concepts in understanding how plants evolve and respond to stress (Willey, 2016). There is, in fact, significant redundancy and/or degeneracy at many levels of plant biological organization including physiological, individual, community and population. Thus, there is a particular need in radiation biology for controlled studies of the effects of chronic low-level irradiation on the phenotypes of groups of plants. Here we aim to investigate the effects on some developmental and morphological phenotypes of chronic low-level irradiation on *Arabidopsis* plants grown for five generations at dose rates of *c.* 40 μGy/h from ^137^Cs. We show that under controlled laboratory conditions there are, overall, no long-term effects over five generations of chronic exposure in the middle of the DCRL ranges for plants.

## Materials and Methods

### Plant Material and Growth Conditions

*Arabidopsis thaliana* Columbia (Col-0), obtained from the Nottingham Arabidopsis Stock Centre (NASC) in the United Kingdom, was grown from seed for its entire growth cycle in Levington’s F2S compost in a growth cabinet (Panasonic MLR-352/352H) with day length of 16 h and night of 8 h at 22°C, 70% humidity (reduced to 50% humidity once siliques had been formed) and *c.*150 μEm^–2^s^–1^ photosynthetically active radiation (PAR). Water was added every 1–3 days as necessary until seed set, when watering ceased. Plants were grown, seed to seed, for 7 generations from a single initial stock. After harvest, each generation of seeds was stored in 1.5 ml Eppendorf tubes and transferred immediately to a refrigerator and kept at 4°C. The first two generations (‘G1’, ‘G2’) were untreated but plants of generation 3 (G3) were grown in a single container to which 90 kBq ^137^Cs had been added. ^137^Cs was obtained from Polatom (05–400 Otwock Weirk, Poland) as a 337 MBq cm^–3^ stock in the form of CsCl in 0.1M HCL solution from which aliquots were taken, and diluted in 500 mL distilled water, to mix thoroughly, carrier free, with 1.5 L dry compost. γ-measurements of treated compost showed that activity dispensed for each generation varied slightly (G3 = 90 kBq, G4 = 90 kBq, G5 = 75.3 kBq, G6 = 107 kBq, G7 = 107 kBq). In generations 4–7, nine white opaque polyethylene containers measuring 18 × 26 × 6 cm were used to grow plants (*n* = 6) in each container. Plants in three containers were negative controls (just Levington’s F2S), three were positive controls (Levington’s F2S + CdCl_2_) and three were treatment (Levingtons F2S + ^137^Cs). This gave 54 plants in total in each generation, with three true replicates of each treatment, each of which consisted of six biological replicates. CdCl_2_ was added to each positive control container as 500 mL of 10μM CdCl_2_ – a Cd addition previously found to affect the development and morphology of *A. thaliana* (Keunen et al., 2011).

**FIGURE 1:**
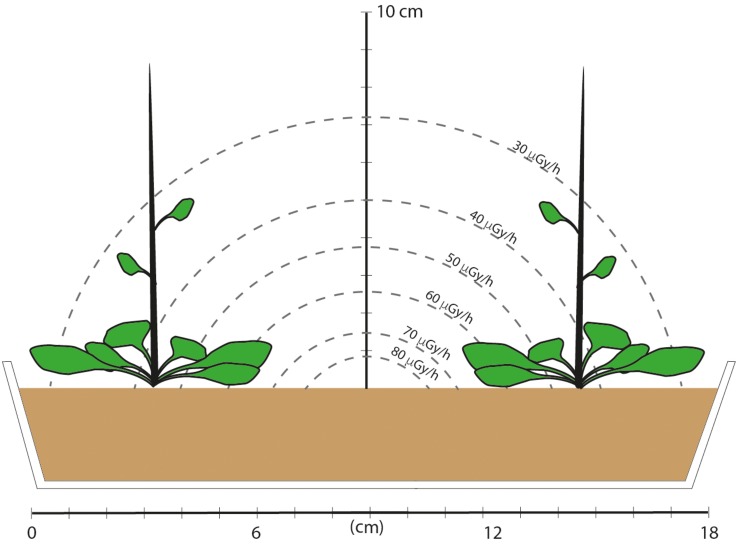
A schematic of the dose field and cross section of the experimental set-up in which plants were grown for five generations. The tray was 10 cm wide and 26 cm long and there were three rows, each with two plants. An estimate of the dose rate field was made based on emission energies from ^137^Cs, and assuming a disk of compost with an area of 375 cm2, a depth of 4.3 cm, 100 kBq of evenly distributed ^137^Cs (an activity representing the middle of the range of those used in the experiment) and take account of attenuation. Assuming a rectangle rather than a disk necessitates complex calculations and it is the range of values rather than specific values that are relevant. These calculations show that plants at the corners, and the leaves at the sides and up the stem therefore had lower dose rates than those toward the center of the tray. Final plant height was about 30 cm after about 60 days, but the majority of leaves remained within 2 or 3 cm of the compost surface. This provided plant material that had experienced a mean dose rate in about the middle of the DCRL dose rate ranges for representative plants.

Seeds were planted directly into the pre-prepared compost of each treatment and excess seedlings were removed, as carefully as possible, at the cotyledon stage to leave six evenly spaced seedlings. There were three shelves in the growth cabinet and containers were moved, in their treatment blocks, to different shelves approximately every 10 days of each growth cycle. Lead shielding (4 cm deep) was installed between each shelf and monitors detected no more than background radiation on the control shelves. This provided plant material that had been grown continuously for five generations in chronic low-level β/γ radiation from ^137^Cs at a known dose rate. Dose rate calculations (Supplementary Data Spreadsheet S1) show that the leaves of the plants were located in an average external dose rate of about 40 μG/h, although some parts of the plants experienced higher or lower doses than this (Figure 1). The activity of ^137^Cs in harvested leaves was undetectable or very low and did not produce internal dose rates that added significantly to the external dose. Sub-samples of some seeds and leaves were occasionally taken to assess germination, root development, redox status and DNA-methylation but the majority of seeds and plants were used for replanting and to assess developmental and morphological endpoints.

### Plant Development

**FIGURE 2:**
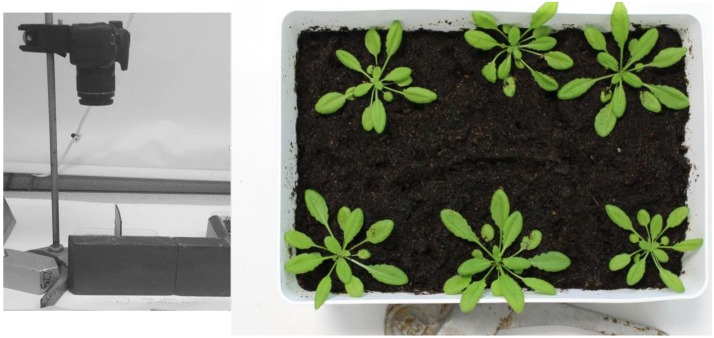
Photographic set-up for recording growth stages of *Arabidopsis* development. **(A)** Camera on mount above location to which trays were moved from the growth cabinet. **(B)** An example of a photograph taken from directly above growing *Arabidopsis* plants.

A digital SLR camera (Canon 1200D) was set up on a bracket (Manfrotto 035C Universal Super Clamp) and pointed downwards against a plain white background (Figure 2A). Approximately every 2–5 days photographs were taken (at a focal length of 24 mm and resolution of 5,184 × 3,456 pixels) from directly above each of the nine containers of growing plants until the end of seven generations of growth (Figure 2B). Digital images were inspected on the computer for individual growth stages of Boyes et al. (2001) and plotted against time.

### Leaf Morphology

Depending on the final number produced, 10–20 leaves from each *A. thaliana* plant from all treatments of generations 4–7 inclusive were harvested at the end of the growth period (i.e., after seeds had been produced). Leaves were then scanned on a Doxie Flip Cordless HD Scanner with the lid closed on the specimens to provide images with a consistent illumination and background. Very small leaves (<0.5 cm diameter) were not scanned. Leaves were spaced apart on the scanner so that they did not come into contact with each other. Leaves were sometimes split during flattening under the scanner lid but if this was not too severe it did not affect final analysis of the traits chosen. Naturally folded leaves occurred on some plants and were included in scans only if they could be unfolded without excess damage. Each scan of leaves produced a high-definition, RGB color digital image in JPEG format. Scanned images were used to analyze leaf morphology using the LAMINA (Leaf shApe deterMINAtion) software developed by Bylesjö et al. (2008). The version used was the Windows 10 Education Edition. LAMINA can determine leaf shape (blade dimensions), area, serrations, holes, circularity and symmetry. The two key endpoints chosen for analysis of *A. thaliana* leaves were leaf area and vertical symmetry. Vertical symmetry is calculated by dividing the length of the 25% vertical area line and the 75% vertical area line (from the left-hand side) of a single leaf. This measure of leaf symmetry has established use in toxicology studies (Alves-Silvia et al., 2018), in which an increase in asymmetry (which reflects developmental instability) is a commonly agreed indicator of stress (Kozlov and Zvereva, 2015). Petioles were removed to expedite leaf symmetry measurements (Nathaniel Street, Umea University, *pers comm*), in Generation 4 this was done digitally but in subsequent generations petioles were removed with a knife before scanning. All images were handled in Adobe Photoshop CC2017 and magnification, aspect ratios, coloring and sharpness were kept constant.

**FIGURE 3:**
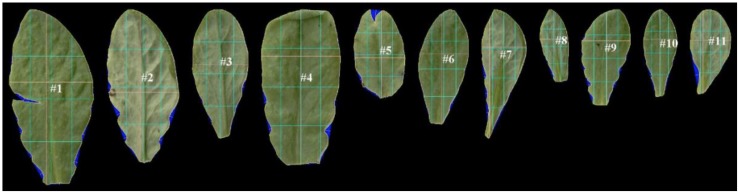
An example of scanned leaf images after processing by LAMINA software (Bylesjö et al., 2008). LAMINA ID tags (e.g. #1) and overlaid grids (colored lines) were used to calculate leaf area and leaf vertical symmetry endpoints. The blue false coloring identifies indents deleted from area calculations. The three light-blue vertical lines show the vertical midline in terms of area (middle vertical light-blue line) and 25% of the total area measured from the left (left-hand vertical line) and 75% of the area measured from the left (right-hand line). The three light-blue horizontal lines show the horizontal midline in terms of area (middle horizontal light-blue line) and 25% of the total area measured from the bottom of the leaf (lower horizontal light-blue vertical line) and 75% of the area measured from the bottom of the leaf (upper horizontal light-blue line). The yellow lines show the widest and longest leaf line (which in some instances coincide with the blue division lines).

After uploading of images, LAMINA identified leaf pixels, overlaid digital grids and performed calculations for each endpoint. This was achieved through sequenced steps, five of which underpin calculations for the chosen endpoints: (1) Thresholding (candidate leaf pixels were identified by leaf color), (2) Segmentation (groups of pixels were identified as leaf and defined as a single object), (3) Filtering (artifacts were removed (checked manually), (4) Object boundary identification (calculation of distances within the leaf ‘object’), (5) Identification of indents (calculation of depths between points identified on the boundary surface). LAMINA outputs were an image file from each scan (Figure 3) and a ‘csv’ file with numerical data.

### Seed Germination and Root Development

A sample of unsterilized seeds from each generation of plants was placed onto strips of damp germination paper (Anchor Paper Co) in petri dishes and kept for 36 h in a growth cabinet with environmental settings for plant growth as described above. Each petri dish was photographed after 36 h with a digital camera and the count function of ImageJ used (with a grid overlaid to prevent double counting) to record germinated and ungerminated seeds in each image.

For analysis of root growth, plants of *A. thaliana* from each experimental treatment were grown for 10 days in half-strength MS agar in square petri dishes. Seeds were washed for 2 min in 70% ethanol and 50% NaClO. They were rinsed four times with sterile deionised water and placed in agar in a line 2 cm from the edge of the petri dish. After germination, containers were placed vertically and wrapped in foil so that only the tops of the petri dishes received light, and the roots could grow downwards with gravity. The plates were sealed with Parafilm^®^ to ensure the growth media remained free of contamination while not jeopardizing gas exchange. One seed from each generation of a treatment group was placed in a row. Each row was scored horizontally across the agar with a sterile blade to ensure accurate placement of seeds. After 10 days petri dishes were removed from the growth cabinet. Lids were removed and the petri dishes were photographed top–down using the camera and bracket described in the previous section, against a dark colored background to offset the light color of the roots. A scale was included in each image. Root analysis was carried out using the Java freeware, ImageJ, which allows for the manual tracing of roots and calculates length along a specified scale (a ruler was included in each image). Only Generation 4 produced roots in each treatment group.

### Redox Status and DNA Methylation

Leaf redox status and the effect of dose rate on it was determined by measurement of GSH concentration and input to the model of Smith et al. (2012) which uses GSH concentration to predict redox status. A sample *A. thaliana* leaf from four plants in each generation and treatment group were snap frozen in liquid nitrogen immediately after excision and stored at −80°C. Because of the scarcity of material following other analyses, plants from all generations were pooled but treatments kept separate. The Sigma Aldrich (CS0260) GSH assay was used but with some alterations of concentration for use with plant extracts (Sigma Aldrich *pers comm*). Frozen leaves were ground to a fine powder in a pestle and mortar chilled in liquid nitrogen. Liquid nitrogen was added to the sample frequently to avoid thawing from heat generated through the grinding process. One hundred mg of powder was used from each sample, mixed with 0.3 ml 5% 5-sulfosalicylic acid (SSA) to clear the sample of other proteins, centrifuged at 18,000 RPM for 10 min in a temperature-controlled centrifuge and the supernatant used for the analyses. Ten μL of each sample supernatant was used in a 96-well plate kinetic read assay. The measurement of GSH was based on a kinetic assay in which catalytic amounts (nmoles) of GSH cause a continuous reduction of 5,5′-dithiobis (2-nitrobenzoic acid) (DTNB) to TNB and the GSSG formed is recycled by glutathione reductase and NADPH. The reaction rate is proportional to the concentration of glutathione up to 2 μM. TNB was measured spectro-photometrically at 412 nm. The assay uses a standard curve of reduced glutathione to determine the amount of glutathione in the biological sample. As the concentrations of the plant samples was low to very low the sample was not diluted before analysis. The GSH standards were also modified to a maximum of 25 μM.

Seeds from plants of *A. thaliana* grown as negative control, positive control and Cs-137 exposed for two generations, i.e., from generation 4, were sent to the Belgian Nuclear Research Centre SCK-CEN in Mol, Belgium. Nineteen plants were grown using a protocol from Vanhoudt et al. (2014) under control conditions for 3 weeks and DNA methylation rates were analyzed using methods described in Volkova et al. (2018).

### Statistical Analyses

All statistical tests were performed in R^1^ using the RStudio graphical user interface^2^. The version of R used in this project [v 3.3.2 (2016-10-31)] was obtained from the CRAN mirror site: https://www.stats.bris.ac.uk/R/. Each set of data was tested for normality using the Shapiro–Wilkes test and Bartlett’s test was used to test for equality of variance between treatments. The Kruskall–Wallace tests were used to test for differences between treatments and generations when data were non-parametric (days to Boyes growth stages in development analyses) and Analyses of Variance (ANOVA) were used for these analyses when data were parametric (leaf area, vertical symmetry, DNA methylation). If a significant difference was detected between treatment means, then Welch’s test was used to identify which treatments were different. A significance level of 0.05 was used for each test.

## Results

### Plant Development

**FIGURE 4:**
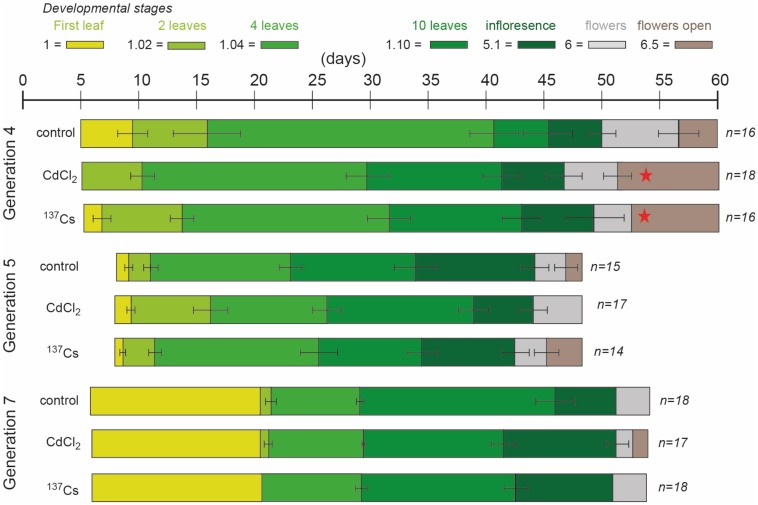
The effect of chronic low-level exposure to ionizing radiation on the development of *Arabidopsis*. Control plants were grown in Levington’s F2S compost. Positive control plants were grown in compost with 10 μM CdCl_2_, plants exposed to ionizing radiation were grown in compost with an average of 94 kBq^137^Cs/kg (dose rate *c.*40 μGy h-1). The developmental phases (colored blocks), determined from photographs, were defined as per Boyes et al. (2001). In different generations the photographs were taken on slightly different days, accounting for some of the differences in days to developmental stage between generations. Ionizing radiation treatment was initiated in generation 3, so plants in generation 4 were in their 2nd generation of exposure. No photographs were taken in generation 6 but they were exposed to the same treatments. Seeds of control and treated plants were harvested, replanted and subject to the same treatment, generation after generation. All treatments in all generations started as *n* = 18 but not all plants survived to set seed, so *n* sometimes differed at the end of each generation. The error bars show the range of days the stage was observed. When the stage was on the same day in all plants there is no error bar. *Indicates a statistically significant difference to control.

There were differences between generations in the average time at which developmental milestones were reached, with generation 5 reaching them faster than the other generations (Figure 4 and Supplementary Data Spreadsheet S2). In the early stages of development this was in part because of slightly different days on which photographs were taken but, ultimately, G5 flowered significantly earlier than G4 and G7. The reasons for this are not clear – experimental conditions were as for the other generations but there were, perhaps, some subtle differences in growth conditions. Within the generations there were some differences between treatments in generation 4 at developmental stages 1.10 (*F* = 13.33, *P* < 0.01) and 5.1 (*F* = 3.96, *P* = 0.03), which occurred significantly earlier in CdCl_2_ and ^137^Cs exposed plants than in control plants (Figure 4). With this exception, in each generation there were no significant differences between treatments in the rate of development. There were no multi-generational trends induced by the treatments, with ^137^Cs exposed plants in G7 being developmentally indistinguishable from control plants. In each generation, after thinning to leave 3 × 6 plants for each treatment, some individuals died before flowering. For each generation the final number of plants were (for control, positive control and IR-exposed respectively) G4 = 16, 18, 16 plants, G5 = 15, 17, 14 plants, and G7 = 18, 17, 18 plants. Thus, 5 generations of growth in a chronic low dose of ionizing radiation of *c.*40 μGy/h had not significantly changed the times at which *A. thaliana* reached key developmental milestones and had not decreased the survival rate of the plants. Overall, it is important to note that in this long-term experiment there was some fluctuation in the developmental milestones between generations and between treatments within a generation. Hence, an investigation of differences between treatments in a single generation or of differences between two generations could have found significant effects that are not maintained in the long-term across multiple generations. It is notable that the absence of effects after G4 is consistent with recovery from an initial effect. We suggest that the key finding of this analysis is that after five generations at *c.*40 μGy/h there were no overall effects on the development and survival of *A. thaliana* under these controlled conditions.

### Leaf Morphology

**FIGURE 5:**
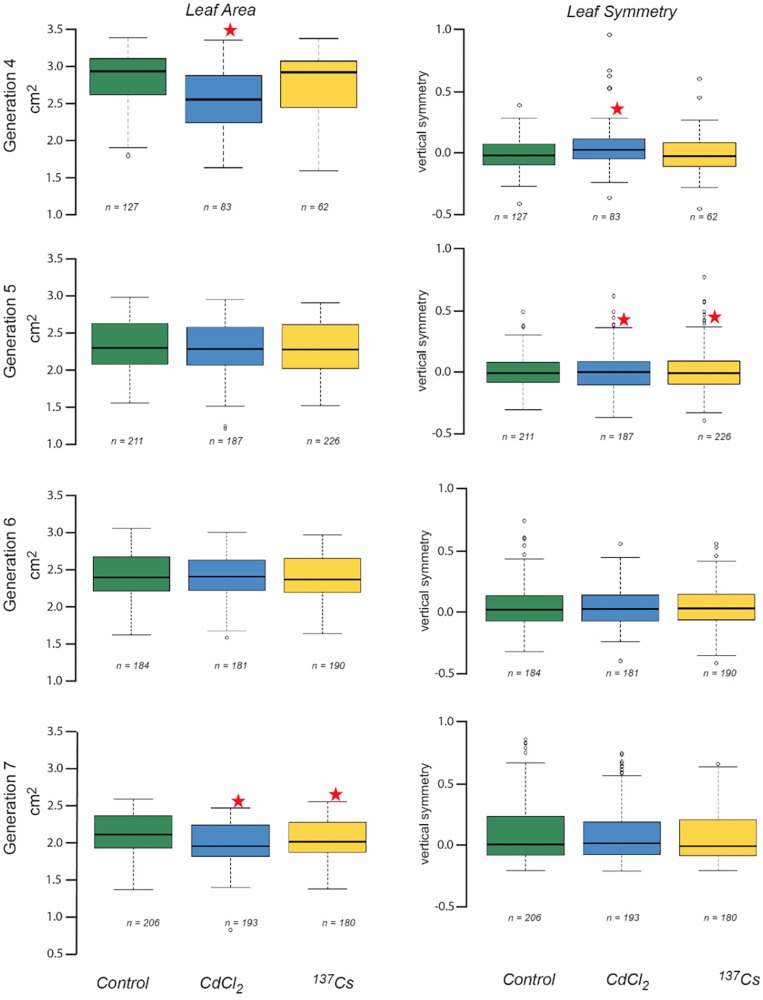
The effect of chronic low-level exposure to ionizing radiation on the leaves of *Arabidopsis*. Controls plants were grown in Levington’s F2S compost. Positive control plants were grown in compost with 10 μM CdCl_2_, plants exposed to ionizing radiation were grown in compost with an average of 94 kBq^137^Cs/kg (dose rate *c.*40 μGy h-1). Boxplots shown with median (thick black line), box for 2nd and third quartile and whisker for 1st and 4th quartile. Outliers are shown as individual circles. * Indicates a statistically significant difference to control.

Leaf area measurements were normally distributed, had statistically significantly different mean values between generations and, in some generations, statistically significant differences between treatments (Figure 5 and Supplementary Data Spreadsheet S3). In G4 the leaves of positive controls had a significantly lower area than the other two treatments, and in G7 there was a significant difference between the negative and positive control, and between the negative control and the IR exposed plants. These differences, though significant, were of small magnitude. Overall, there were no long-term trends in the data, although it is notable that in G7 the CdCl_2_ and ^137^Cs exposed plants were healthy and although they developed at the same rate as the control plants had a slightly reduced area.

Datasets of vertical symmetry of the leaves of plants were not always normally distributed and were ln-transformed before analysis if necessary. The vertical symmetry of leaves from different generations and treatments was always very close to zero (i.e., the leaves were, on average, symmetrical) and although it varied by only small amounts, there were statistically significant differences in some instances (Figure 5). In G4 the positive control plant leaves were slightly asymmetric as compared to those of the other treatments, and in G5 both CdCl_2_ and ^137^Cs exposed plants were slightly asymmetric as compared to controls. In both these instances this was because there were a number of leaves with a positively skewed asymmetry in the statistically different treatment groups. The number of leaves suitable for scanning varied between generations and treatments and although it was not intended as an endpoint it is notable that there were no significant long-term trends in leaf numbers for scanning across the generations. Overall, although there were sometimes some differences between generations and/or treatments in leaf vertical symmetry there were no significant long-term trends associated with exposure to *c.*40 μGy/h IR over 5 generations.

### Seed Germination and Early Root Growth

**FIGURE 6:**
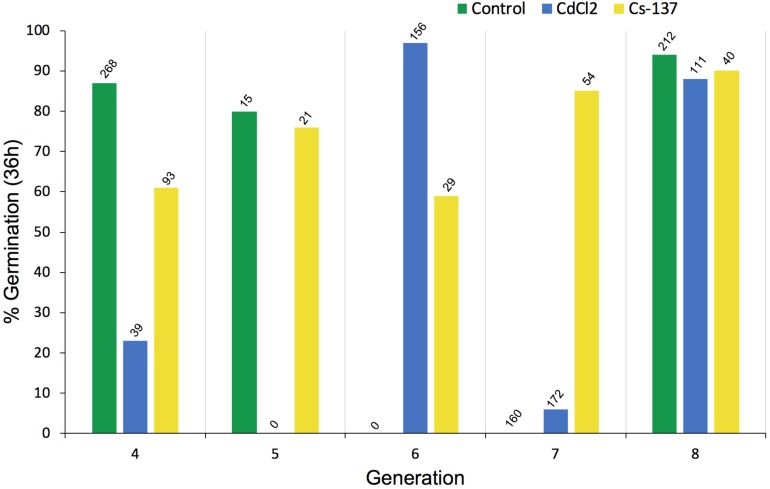
The effect of chronic exposure to ionizing radiation on germination of *Arabidopsis thaliana* seeds over multiple generations. Plants were exposed during their growth but not during the germination test to chronic low-level irradiation at c. 40 μGy/h from Cs-137 starting in generation 3, i.e., the plants that produced the seeds tested in generation 4 above had been exposed to IR for their entire life span in the previous generation. The seeds in generation 8 above had been exposed for their entire life-span during generations 3–7. The total number of seeds used in each germination test is given above each bar. At harvest, from which a variable amount of seed was collected, seeds were first allocated for planting the next generation so the differences in seed numbers above depended primarily on how many seeds remained for the test. CdCl_2_ was in the growth medium of the plants but not during the germination test.

The number of seeds available for germination tests and root growth tests differed across treatments and generations because seeds from each generation were used, in the first instance, for replanting the next generation of plants – those that happened to remain were used for the tests reported in this section. Total seed production was not one of the key endpoints chosen for the experiments reported here so it was not measured and the numbers in Figure 6 do not reflect total seed production. The germination tests using some seeds from each generation of chronic exposure to IR at *c.*40 μG/y did not, however, reveal any long-term trends in % germination (Figure 6). Notably, the % germination of seeds harvested from generation 7 plants (‘generation 8’ in Figure 6) was high in all the treatments and, it seems clear that if a single generation is investigated it is possible to get differences between treatments although there was no overall trend across generations.

**FIGURE 7:**
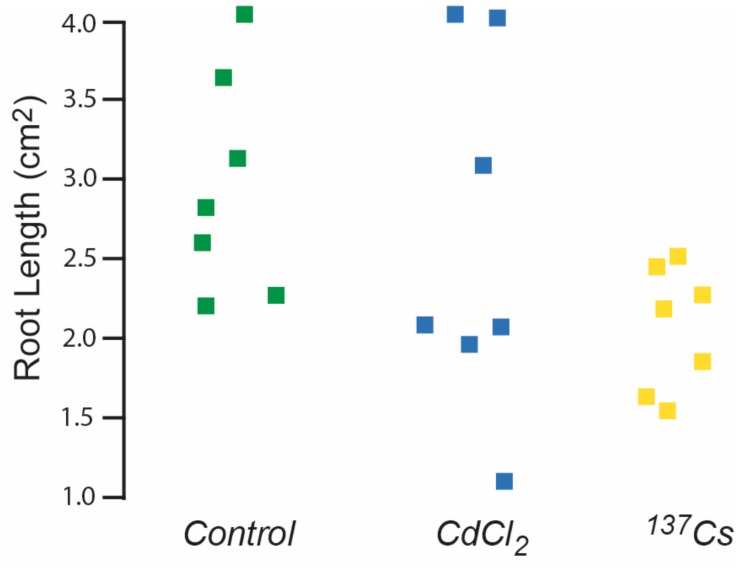
The effect of ionizing radiation of the growth of roots 10 days post-germination. Seeds were from plants chronically exposed, in the growth medium, to 10 μM CdCl_2_ or ionizing radiation of c.40 μG/h from Cs-137 for two generations. Kruskal–Wallis tests indicated no significant difference between the treatments.

Seeds remaining after generational replanting and germination tests were used to investigate root growth. Generation 4, i.e., those that had received chronic irradiation for two generations, were the only ones in which a full set of root data from all treatments was obtained. Even though after 10 days the mean root length produced by seeds from plants previously exposed to ionizing radiation was less than that of other treatments there were no statistically significant differences in root length (Figure 7). These data, and those from the germination tests outlined above, do not preclude there being some effects on root growth in particular but do not indicate significant effects of exposure to *c*.40 μG/y from Cs-137 on long-term germination rates. They emphasize that even under controlled conditions these traits can be quite variable and that quite large numbers of samples and multiple generations are necessary to detect long-term effects at the dose rates used here.

### Redox Status

**FIGURE 8:**
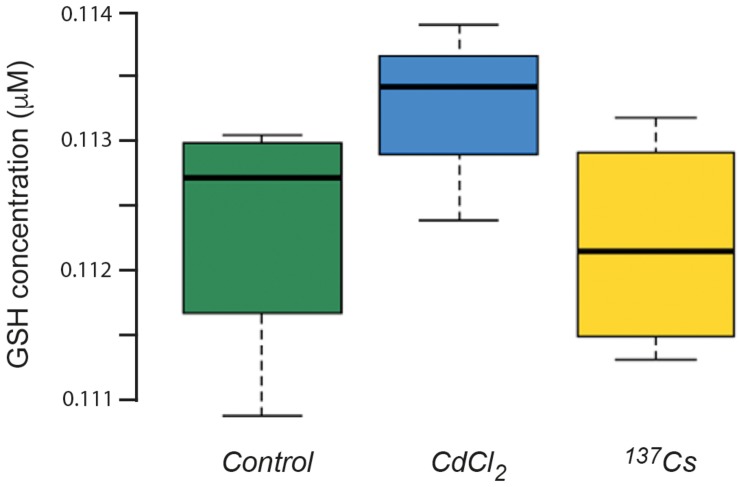
The concentration of glutathione (GSH) in plants exposed to CdCl_2_ or ^137^Cs for 5 generations. Measurements were performed on leaves collected from each generation and pooled for analysis. (Control *n* = 12, CdCl_2_ *n* = 12, _137_Cs *n* = 24).

The mean concentration of GSH in leaves pooled from the all generations of *A. thaliana* was about 112 nM, with no statistically significant difference between treatments (Figure 8). This is a much lower GSH concentration than is usually found in plant leaves under optimal conditions, for which 300 nmol/g fresh weight is more usual (Noctor et al., 2012). Although the similar values for control and treated plants suggest that this low value is due to extraction or analysis protocols, we tested using the model of Smith et al. (2012) whether it could theoretically have been produced by IR exposure used here. This model uses the known production of ROS from radiolysis induced by a particular radioisotope (in this case ^137^Cs) and, assuming that all the ROS react with GSH as a single anti-oxidant and that the GSH/GSSG ratio determines redox poise, predicts the concentration of GSH and redox poise over time at a given dose rate. The model is dynamic and accounts for the replenishment of GSH.

**FIGURE 9:**
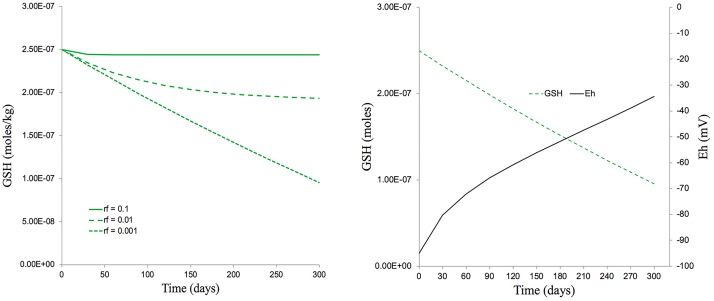
The predicted effects of chronic ionizing radiation exposure on GSH concentration and redox status (E_h_) in plants exposed to a dose rate of *c.*40 μGy/h from _137_Cs for five generations (300 days). **(A)** A set of scenarios, one of which [a replenishment rate of GSH of 0.1% per day (rf = 0.001)] gives a final GSH concentration of 100 nM after 300 days exposure – approximately the concentration measured in our multi-generational experiment. **(B)** A calculation, using the Nernst equation, of changes in E_h_ that would occur over 300 days under the scenario in A which produces a final GSH concentration of 100 nM. This measured concentration and replenishment rate of GSH used in the model (100 nM and 0.1% respectively) are low compared to those likely to be found in most plants. The calculations used the model for Cs-137 of Smith et al. (2012) which assumes that there are no anti-oxidants other than GSH, that the radiolysis products induced by Cs-137 all directly reduce GSH and that there is replenishment of GSH. Calculations are in Supplementary Data Spreadsheet S4.

If we take the approximate timespan (300 days) of the 5 generations exposed to IR in the experiments reported here, then, assuming there are no other anti-oxidants and an extremely low replenishment rate of 0.001 GSH per day, a dose rate of *c.*40 μGy/h could have reduced a GSH concentration of 250 nmoles/kg down to about 110 nmoles/kg over 300 days (Figure 9). This, theoretically, could change the average E*_*h*_* from −90 mV to −40 mV, a change of redox poise likely to have caused significant stress to the plants (Figure 9). With replenishment rates of 0.01 or 0.1 (which are much more realistic) a dose rate of *c.*40 μGy/h from ^137^Cs has essentially no effect on redox poise and, in reality, the antioxidants system of plants has many other components. So, overall, the ROS production rate from a dose rate of *c.*40 μGy/h seems very unlikely to have reduced the GSH concentration to *c.*110 nmoles/kg and, given the GSH concentrations that usually exist in plants, their replenishment rates and the existence of additional anti-oxidants, seems very unlikely to have produced a change in redox poise that might have caused redox ‘stress’ to the plants in the experiment reported here.

### DNA Methylation

**FIGURE 10:**
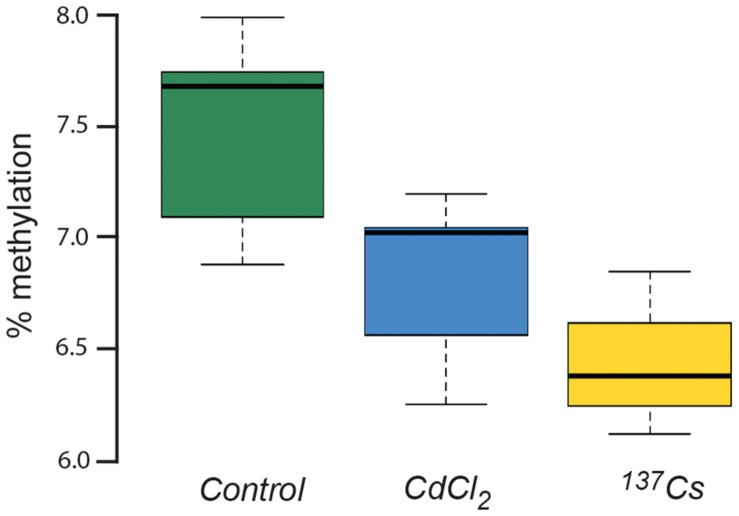
The % methylation of DNA extracted from leaves of plants chronically exposed, in the growth medium, to CdCl_2_ at 10 μM and to ionizing radiation from Cs-137 at a dose rate of *c.*40 μG/y for two generations. ANOVA showed a statistically significant difference between treatments, with both CdCl_2_ and Cs-137 exposed plants being significantly different to the control plants.

DNA in plants is known to be quite highly methylated compared to other organisms, with rates of up to 10–15% being reported in *A. thaliana* (Mirouze and Vitte, 2014). This rate of methylation is consistent with that shown in Figure 10, which also shows that there was a significant difference in DNA methylation rate between treatments after two generations of exposure at *c*.40 μGy/h. Depending on the position in the genome at which they occur and the genetic context in which they occur, changes in DNA-methylation can have contrasting effects. Environmental stressors such as heat (Liu et al., 2018) or salinity (Al-Harrasi et al., 2018) can increase or decrease the methylation of DNA so it is not appropriate to conclude that the changes induced by IR have ‘stressed’ the plants in our experiments, but it seems likely that they have induced changes in gene expression and, perhaps, therefore in physiology. These changes do not seem to be associated with changes in the developmental and morphological traits measured here but are, perhaps, consistent with other genetic and physiological changes that have been reported in plants after exposure to chronic low-level IR.

## Discussion

In each treatment of the experiments reported, the plants constitute a small population that was followed across 7 generations, with traits measured on them at the developmental, morphological and physiological levels. The results therefore provide new insights into the effects of chronic low-level IR-exposure at different biological levels over a number of generations under controlled conditions. Overall, the results suggest that there were, under the conditions used and based on the traits measured, no consistent multi-generational effects at the population level. This is not to say that there are no effects of chronic exposure to low-level IR at the dose rates used. At particular points across the generations there were differences in development, leaf area, leaf symmetry and DNA methylation in the IR exposed treatments. The traits we measured were variable, which means that ascribing the effects to IR must be done with caution but also that some subtle effects of IR may be hidden. It is also possible that differences might be produced only after even more generations of exposure. A biological perspective is necessary to understand how there might be effects on some measured traits but that this is not significant at the population level. Here we use a biological perspective to provide new evidence that the current DCRLs recommended by the ICRP probably do protect, at least to the extent legally required, flora from the effects of IR and suggest how such protection might be given with greater confidence. The data and the suggestions we make are also consistent with the conclusions of UNSCEAR and the ERICA project.

In the results reported here, there were instances in particular generations when plants exposed to IR at *c*.40 μGy/h were developmentally or morphologically statistically significantly different to one or more of the control groups. In all of these instances, if a single generation of plants had been investigated a conclusion of significant effects of IR on plant traits could have been made. However, measuring traits for multiple generations has shown that at this dose rate there were no long-term ‘systematic’ effects over the generations on development or morphology of exposure to IR at *c*.40 μGy/h or at least no differences that were detectable over and above the variation of the traits. It is, however, notable that after 5 generations of IR exposure plants are, on the basis of traits we measured, developmentally indistinguishable and morphologically distinguishable only by a very small difference in leaf area, from control plants. It is possible that some of the differences in effects between treatments in particular generations were induced by IR or CdCl_2_ exposure – low-intensity exposure to environmental stressors can make development less stable – but given that the measured traits in our populations of plants were at least as variable between generations within a single population (treatment) over the generations as they were between the different populations (treatments) in a particular generation it is, perhaps, as likely that variability not associated with the experimental treatments has produced instances in which there are significant differences between treatments. Thus, for the traits we measured under controlled conditions we found no significant long-term differences induced in *A. thaliana* at the population level by exposure to IR at *c*.40 μGy/h. Overall, the results reveal the extent to which some developmental and morphological traits used as ‘end-points’ in radioecological effects studies can vary between populations and generations – an important point to remember when only investigating particular populations or generations.

There is, however, some evidence that chronic exposure to IR might have had physiological effects. Although the data were sparse for redox poise and the GSH concentrations were low and there was no statistically significant difference between the negative controls and the IR-exposed plants there are indications of physiological effects. The total number of oxidative radicals produced by *c.* 40 μG/h from ^137^Cs is low compared to the number that will be being produced during the physiological activity of the plant (Smith et al., 2012), so effects of ROS directly produced by radiolysis would be surprising but there was a statistically significant difference in DNA methylation, which was decreased by exposure to IR. The exact physiological significance of this change was not explored, and the effects of methylation depend on which sequences and which genes experience changes in methylation, but differences in DNA-methylation are likely to reflect physiological effects and/or have physiological consequences. Previous reports have shown differences in DNA methylation and cytogenetic changes induced in plants at similar IR exposure rates (Kovalchuk et al., 2003) and changes in DNA methylation have been reported to have significant physiological consequences in other contexts (Liu et al., 2018). The results reported here are, therefore, consistent with other suggestions that changes at sub-cellular levels of biological organization, e.g., cytogenetic and physiological, can be induced at the dose rates used here.

A trait, such as those measured here and those often used as endpoints in toxicological studies, is an inherent property of an organism whereas a function is a relational process, i.e., it is dependent on context, both the biological and environmental context (Farnsworth et al., 2017). Traits can be functionally redundant (defined as two or more instances of the trait, each with the same function, and any of which can therefore perform the function) or degenerate (defined as different traits performing the same function). Environmental regulatory efforts, at least in the first instance, usually focus on impacts that affect function at the level of individual, population or community. A useful way of conceptualizing living systems is as autocatalytic nested hierarchies that function across biological levels (Farnsworth et al., 2017). Function at a given level is contextualized by the next level in the hierarchy and by the environment. Across biological levels, redundancy and degeneracy in the relationship between traits and function are not only quite common but are probably a significant adaptation, because of the resilience and adaptability they confer, of organisms to a variable environment. It has been suggested that because they are sessile this is particularly the case for plants (Willey, 2016). Thus, there might be, and can be, effects of IR on traits at one biological level that do not necessarily affect function at the next level of the hierarchy. We suggest that the data we present here, and other data previously published, suggest that DCRLs for plants span dose rate ranges in which some *traits* might be affected at lower levels of biological organization but that at the higher levels of organization, in particular those that produce the phenotypes on which survival depends, *function* is not affected to the extent that adverse impacts on a population are expected.

The suggestions above are based on results from one species but it has been known for several decades that different plant species differ significantly in their susceptibility to IR (e.g., Woodwell, 1967). In general, herbaceous species are considered less sensitive than woody species, with deciduous woody species being less sensitive than coniferous woody species. Investigations of the long-term effects of short-term high-dose γ-exposure of vegetation at Brookhaven National Laboratory suggested that *Carex pensylvanica* was particularly radioresistant, which was attributed to its rhizomatous habit (Stalter and Kincaid, 2009). As it is herbaceous, *A. thaliana* is probably relatively radioresistant, so for woody species that are significantly more sensitive than *A. thaliana* it is possible that *c*.40 μGy/h might produce effects on function that are significant at the population level. The radioresistance of rhizomatous species is usually attributed to growing points on their rhizomes not being as exposed as above ground shoots. This would only apply to above-ground exposure rather than to exposure from contaminated soil, so for chronic exposure from the soil *A. thaliana* is likely to be quite radioresistant. It is not clear why plant species differ in their radiosensitivity. It has long been suggested that plant radiosensitivity correlates with nuclear volume (Sparrow and Miksche, 1961; Sparrow and Woodwell, 1962), often specifically the interphase chromosomal volume. There are, however, exceptions to this and it has been suggested (Woodwell, 1967) that a more useful generalization is that radiosensitivity correlates with the proportion of photosynthetic material a species has. Jordan (1986) noted the ecological foundations of this suggestion, given the differences in life cycle, investment in particular organs and so on, that link to it. There are, therefore, grounds for believing that as a fast cycling herb with a small chromosomal volume and a high proportion of photosynthetic material, *A. thaliana* will be radioresistant compared to many plants and that at the moment there is a case for regulators to categorize some species as being in a more sensitive category, as it currently the case for pine trees.

The plants grown in the experiments reported here experienced growing conditions unlike those experienced by plants growing in the field. For our experimental plants most of the environmental factors that affect plant growth were essentially constant or changed between two constant states (e.g., light/dark) and the plants were not exposed, as far as could be ascertained, to significant herbivory or disease. The growth conditions are unlikely to have been entirely optimal for growth but might have been approaching such conditions – although it must be remembered that exposure to environmental variation isn’t necessarily ‘stressful’ to plants and, in a real sense, is what plants have evolved to thrive with. The particular growth conditions our plants experienced mean, however, that care must be taken in speculating about the implications of the results reported here for plants in particular field locations. But it is important to note that in contrast to most data reported from the field, the experiments reported here had good sets of positive and negative control plants and, most importantly, the plants experienced a dose rate that can be calculated with reasonable certainty. However, there are ecological grounds, supported by field-lab comparisons, for suggesting that plants in the field subject to other stressors might be more susceptible to IR than plants grown under experimental conditions (Garnier-Laplace et al., 2013).

The empirical basis for discussions of plant sensitivity to IR and the dose rates at which regulation might be necessary are still, however, relatively scant compared to those for other toxins subject to environmental regulation. Cadmium is a plant toxin that occurs naturally at low, though variable, background concentrations in many places and is, with mercury and lead, one of the most problematic metal contaminants in the environment (Willey, 2016). Although each plant toxin or stressor can cause particular symptoms, not only are there physiological effects of Cd that are similar to those of IR [DNA damage (Rani et al., 2014) and oxidative stress (Cuypers et al., 2010)], it can also be useful to environmental regulation to envisage analogous toxicity scenarios. We suggest that the chronic low doses of IR that plants were exposed to in the experiments reported here are analogous to, perhaps, the 1 to 5 mg/kg range of Cd in soil. We added 5 μmoles of Cd (0.560 mg) to 1.2 kg of substrate and found occasional effects. Although the soil concentrations of Cd that are generally considered phytotoxic vary with soil type, species and type of exposure, in general the 1–5 mg/kg is a ‘sub-toxic’ range in which some effects might be found but effects on a population of plants would not generally be expected (though the transfer of Cd up the foodchain might be significant). Concentrations of above 10 mg Cd/kg soil, which are about 50 times a mean background of Cd at 0.2 mg/kg) are often reported to be toxic to plants. This analogy might be helpful – a dose rate of *c*.40 μGy/h is about 50 times background – because there might be some effects though not at the population scale, some species might be sensitive at this dose rate and transfer up the foodchain might be significant.

## Conclusion

In the relatively radioresistant *A. thaliana* grown under controlled conditions over 5 generations with a dose rate of c. 40 μGy/h from ^137^Cs, we did not find effects likely to have significant impacts on function at the population level. The results indicate that effects on traits at lower biological levels in particular are possible at this dose rate and provide novel evidence that the loose association between genotype and phenotype characteristic of plant stress responses buffers the population from functionally adverse impacts. Taking into account the possibility of greater sensitivity of plants growing under field conditions, and the presence of multiple stressors, a biological perspective helps to provide confidence that a DCRL of 4.17 – 41.7 μGy/h for sensitive woody plants and of 41.7–417 μGy/h for less sensitive plants are the ranges for action that will protect plant populations. Really long-term studies of the type that are beginning to now become possible at sites with a history of radioactive contamination are necessary to confirm the suitability of these DCRLs. There are numerous reasons why for environmental protection, agriculture, horticulture, space science and radiobiology it might be useful to understand why plants differ in their sensitivity to IR but given that the risks to populations of plants at DCRL ranges seem very low and that there are significant benefits from the use of radiation, current regulation seems appropriate. Increased confidence in DCRLs could be provided by understanding more about possible effects at the top end of the DRCL range and by understanding species differences in sensitivity to IR.

## Data Availability Statement

All datasets generated for this study are included in the article/Supplementary Material.

## Author Contributions

NC co-designed the experiments, carried them out, and co-wrote the manuscript. AH helped to design and carried out the experiments, and provided the technical support. NW was the PI, conceived the approach, co-designed the experiments, and co-wrote the manuscript.

## Conflict of Interest

The authors declare that the research was conducted in the absence of any commercial or financial relationships that could be construed as a potential conflict of interest.

## Footnotes

1 https://www.r-project.org/

2 https://www.rstudio.com

## Supplementary Material

DATA SPREADSHEET S1 Dose rate Calculations.

DATA SPREADSHEET S2 Growth Stages.

DATA SPREADSHEET S3 Leaf Area.

DATA SPREADSHEET S4 Leaf Symmetry.
